# Applications of Carbon Dots in Optoelectronics

**DOI:** 10.3390/nano11020364

**Published:** 2021-02-01

**Authors:** Evgeniia A. Stepanidenko, Elena V. Ushakova, Anatoly V. Fedorov, Andrey L. Rogach

**Affiliations:** 1 Center of Information Optical Technology, ITMO University, 197101 Saint Petersburg, Russia; stepanidenko.e@mail.ru (E.A.S.); elena.ushakova@itmo.ru (E.V.U.); a_v_fedorov@inbox.ru (A.V.F.); 2 Centre for Functional Photonics (CFP), Department of Materials Science and Engineering, City University of Hong Kong, Kowloon, Hong Kong, China; 3 Shenzhen Research Institute, City University of Hong Kong, Shenzhen 518057, China

**Keywords:** carbon dots, emission, composite materials, solar cells, light emitting diodes

## Abstract

Carbon dots (CDs) are an attractive class of nanomaterials due to the ease of their synthesis, biocompatibility, and superior optical properties. The electronic structure of CDs and hence their optical transitions can be controlled and tuned over a wide spectral range via the choice of precursors, adjustment of the synthetic conditions, and post-synthetic treatment. We summarize recent progress in the synthesis of CDs emitting in different colors in terms of morphology and optical properties of the resulting nanoparticles, with a focus on the synthetic approaches allowing to shift their emission to longer wavelengths. We further consider formation of CD-based composite materials, and review approaches used to prevent aggregation and self-quenching of their emission. We then provide examples of applications of CDs in optoelectronic devices, such as solar cells and light-emitting diodes (LEDs) with a focus on white LEDs.

## 1. Introduction

Alongside well-known light-emitting materials such as organic dyes, rare-earth elements, and semiconductor quantum dots (QDs), carbon-based luminescent nanomaterials such as carbon dots (CDs) are gaining increasing popularity currently [1,2]. CDs are tiny nanoparticles composed of sp^2^- or sp^3^-carbon domains and rich in oxygen- and nitrogen-containing functional groups. According to Refs. [3,4], luminescent carbon nanoparticles can be classified as graphene quantum dots (GQDs), carbon dots (or nanodots), and polymer dots. GQDs are composed of one to several layers of graphene often functionalized with molecular groups at the edges, and possess a quantum-sized effect. CDs are mostly spherical or quasispherical nanoparticles, where the degree of crystallinity can vary over; they often demonstrate excitation-dependent photoluminescence (PL). Polymer dots can be considered as aggregated or cross-linked oligomers/polymers and contain emissive groups within the polymer network. This review will focus on spherical or quasispherical luminescent carbon nanoparticles, which are designated hereafter as CDs. A considerable research interest in these nanoobjects is fueled by the possibility of tuning the CD emission over a wide spectral range, which can be accomplished at the synthetic stage by varying precursors and reaction conditions [5,6,7]. Importantly, CDs do not contain any toxic elements and are considered as non-toxic materials, in a stark contrast to most of the “classical” Cd- or Pb-based semiconductor QDs [8,9]. Moreover, the PL quantum yield (QY) of CDs can easily reach very high values, especially in the blue and green spectral range [10,11]. Being a biocompatible and non-toxic alternative to QDs, CDs have been widely used in bioimaging, sensing, and other biomedical applications [12,13,14,15,16,17,18,19]. Composite materials based on CDs have been often reported to have phosphorescence observed at room temperature, which is a useful property for data encryption [20,21,22]. CDs even found some applications in agriculture, where they were used to improve plant growth and production [23]. Due to their strong absorption [24], CDs were used as sensitizers in solar cells [25,26]. Considering a variety of synthetic methods and precursor materials of CDs and their variable degree of crystallization, the electronic structure and optical processes occurring in CDs may undergo significant transformations depending on their inner structure, chemical composition, and types of molecular moieties at their surface [2,27]. Depending on the chemical composition, the PL of CDs may originate from heteroatom-containing sp^2^-domains [28,29], polycyclic aromatic hydrocarbons [30,31], inclusions of organic dyes [32,33], and surface functional groups [34,35,36]. Luminescent centers can be located both at the surface of CDs or be embedded into a matrix of amorphous carbon within CD cores [37,38]; this opens up a great variety of interactions of CDs with surrounding media, which often include energy or charge transfer processes. Due to the fact that there may be different luminescent centers formed inside CDs during the synthesis, these nanoparticles often possess a wide PL spectrum with excitation-dependent PL band [30]. This is beneficial for their employment in white light emitting diodes (WLEDs) [39,40]. Overall, the implementation of CDs in optoelectronic devices attracted much scientific attention in recent years [41,42,43,44]. For instance, in a recent review [45], possible applications of CDs not only for light emission but also for energy storage have been discussed.

In this review, we will introduce the recent synthetic progress towards multicolor-emissive CDs suitable for application in optoelectronic devices, with a focus on approaches that shift their PL to a longer wavelength, and those that increase their PL QY. We consider methods used for the modification of surface of CDs to adjust their electronic energy levels, and approaches aimed to preserve their attractive PL properties when CDs are embedded into solid matrices, i.e., to prevent aggregation and self-quenching processes. We provide examples of their use in solar cells and LEDs, with the focus on WLEDs, and emphasize the difference between the electroluminescent and down-conversion CD-based WLEDs. The literature analysis provided here shows that CDs are indeed very promising materials for further research and development in terms of the broad field of their optoelectronic applications.

## 2. Improvements of Optical Properties of CDs

### 2.1. Synthesis of Multicolor-Emitting CDs

**Figure 1:**
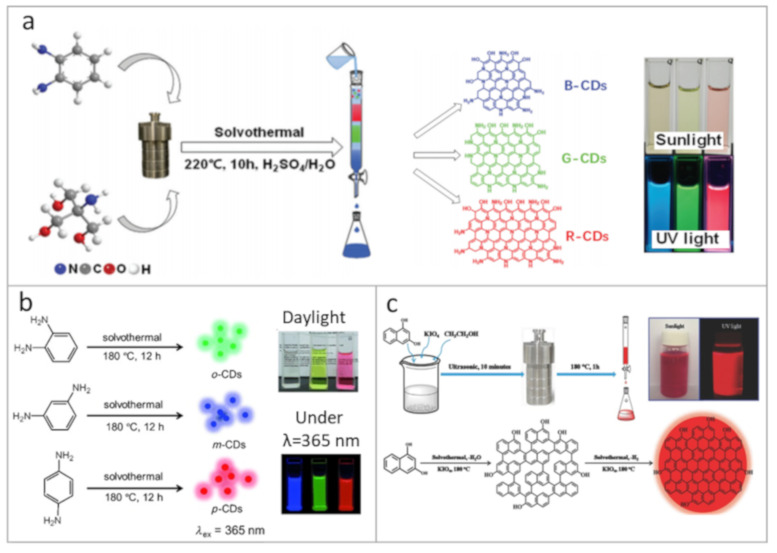
Synthetic approaches towards carbon dots (CDs) with multicolor emission and high photoluminescence (PL) quantum yield (QY), exemplified by respective photographs of CD solutions taken under daylight and UV lamp: (**a**) schematic representation of the one-pot synthesis and separation route for blue, green and red emissive CDs. Reprinted with permission from reference [39], Copyright Royal Society of Chemistry, 2020; (**b**) synthesis of the blue, green, and red emissive CDs from three different phenylenediamine isomers. Reprinted with permission from reference [15], Copyright Wiley-VCH, 2015; (**c**) schematic diagram showing the preparation and growth mechanism of red emissive CDs based on 1,3-dihydroxynaphthalene and KIO_4_ in ethanol. Reprinted with permission from reference [62], Copyright Wiley-VCH, 2017.

It was theoretically shown that the electronic structure of carbon nanoparticles can be governed by size, chemical composition, doping, etc. [19,46,47] In particular, in [48] CDs were considered as nanoparticles with sp^3^-hybridized amorphous carbon cores that contain partially sp^2^-hybridized carbon domains, and it was predicted that the emission of CDs can be redshifted by increase of hybridization factor of those domains within CDs. Another theoretical investigation reported in [49] showed that covalent-bonded dimers of polyaromatic hydrocarbons at the CDs surface, in contrast to non-interacting monomers, result in a redshift and broadening of PL band, with decrease of its intensity. Similar approaches were implemented to develop the variety of CDs synthesis methods. To produce CDs with tunable PL, different approaches can be applied (Figure 1): change of precursors (different type of precursors, molar ratio of precursors, different solvents, etc.); control of chemical reactions (temperature, pressure, time, use of catalysts, pH of the environment, etc.); and interactions with the environment (solvent polarity, controllable aggregation, etc.).

Several studies have shown that under similar synthetic conditions, the molecular structure of isomers [15,50,51,52] and the molar ratio of precursors [51,53,54] have a significant effect on the optical properties of the resulting CDs. In [55], the role of doping heteroatoms was demonstrated: nitrogen- and sulfur-doping resulted in the appearance of additional energy levels, which shifted the emission band of CDs towards longer wavelength. CDs can be doped by metal ions during the synthesis: by adjusting the molar ratio of p-phenylenediamine to ZnCl_2_, the CD emission was tuned from 390 to 610 nm with PL QY of 19% [54]. Theoretical simulations showed that the Zn doping could lead either to red or to blue PL shifts, depending on the functional groups on the CD surface. In [56], it was shown that the PL of CDs could be tuned from the blue to red spectral region via changing the molar ratio of precursors together with the reaction time. It was also shown that with increasing the sp^2^-domain size, a redshift of the PL band could be achieved. Yuan et al. [52] demonstrated the synthesis of multicolor-emitting CDs from citric acid and 2,3- or 1,5-diaminonaphthalene, for which the PL was adjusted by varying reaction times and adding an appropriate amount of concentrated sulfuric acid as a catalyst, leading to the increased dehydration rate. Synthesized CDs had high crystallinity with PL redshift accompanied by a gradual decrease of PL QY, which was explained by the reduction of amino groups at edge sites. Hu et al. [57] showed that the addition of H_3_PO_4_ to the ethylene glycol-based CDs resulted in the PL redshift, which was not observed for the citric acid-based CDs. At the same time, the addition of NaBH_4_ led to a blueshift of the PL peak for both types of CDs. Another way to influence the dehydration of carbon cores can be realized via temperature control. In [53], the change of reaction temperature from 140 to 200 °C resulted in tuning the PL of CDs over a wide spectral range from deep blue to red. Multicolor-emissive CDs could also be synthesized through one-pot synthesis, followed by their separation using column chromatography. This was demonstrated in [39], where CDs made from o-phenylenediamine and tris(hydroxymethyl)aminomethane showed blue, green, or red emission after the size separation procedure (Figure 1a). Since CD emission may also originate from their surface moieties, tuning of the PL band can also be realized by changing the solvent polarity [51,58,59]. According to [56], the PL shift can also be associated with aggregation states of CDs, which is controlled by the environment.

With such large variety of approaches to synthesize CDs, one of the main existing challenges is to be able to increase their PL QY with a simultaneous shift of the PL to longer wavelength. At present, several synthetic methods allowing us to obtain blue-emitting CDs with high PL QY values of 70% or higher can be found in literature [12,18,60]. Blue-emitting CDs with an extremely high PL QY of over 94% have been synthesized by a simple hydrothermal treatment of folic acid [10]. Great success was also achieved in development of synthetic routes towards green or yellow emitting CDs, with PL QY in the range of 45–70% [11,17,50,61]. It was shown that in order to increase the PL QY of both green and yellow CDs, it may be necessary to add trace amounts of HCl during the synthesis, which promotes dehydration of the precursors and carbonization of the molecular moieties, thus leading to formation of CDs with stable luminescent centers [61].

At the same time, the PL QY of red emissive CDs rarely exceeded 25% so far [15,16,52,53,57,58]. In [57], CDs with PL located in the range of 400–710 nm were produced by adjusting synthetic conditions. It was shown that by increasing the oxygen content in CDs, a PL redshift towards 600–700 nm could be achieved. Since solvents may affect the carbonization process, Sun et al. studied their impact on the optical properties of CDs [16]. It was shown that CDs synthesized via a microwave-assisted method from citric acid in formamide have PL at 640 nm (λ_ex_ = 540 nm) with a PL QY of up to 22%. These CDs had a graphite-like core with surface functional groups containing nitrogen and oxygen. Relatively high values of the PL QY (26%) for red-emissive CDs were reported in [15]. The PL redshift was accomplished by the increase of the nitrogen content and CD size simultaneously, which was achieved by using the p-phenylenediamine (Figure 1b). The PL redshift was also reported for CDs synthesized with carboxylic groups at the surface, together with a high degree of the core carbonization [53]. By functionalizing the CD surface with metal ions, PL QY for emission at 580 nm could be enhanced from 6% to 46% [63]. To the best of our knowledge, the highest reported PL QYs of red-emissive CDs reached 53% [51,62]. Wang et al. [62] synthesized CDs by solvothermal treatment of 1,3-dihydroxynaphthalene and KIO_4_ in ethanol and used the approach of sequential dehydration and dehydrogenation resulting in the formation of large-sized sp^2^ domains with -OH groups at the edge sites, which both contributed to the red emission of CDs (Figure 1c). These CDs exhibited high crystallinity and excellent photostability. Lin et al. [51] showed that the CDs hydrothermally synthesized from p-phenylenediamine and dimethylformamide (DMF) might reach PL QY as high as 52%, while their PL position was tunable by the solvent polarity.

Alongside the development of the synthetic routes towards CDs with a high PL QY whose emission is tunable over a wide spectral range, the change of the CDs surface chemistry is one of the routes to further control their optical and electronic properties. This can be realized by the formation of specific molecular groups during the synthesis [64], chemical treatment of CDs with acids or bases [63], and other kinds of post-synthetic treatments [65,66]. It was previously shown by density functional theory (DFT) that the electron-donating/withdrawing nature of surface functional groups affects the positions of HOMO and LUMO levels of CDs [67,68,69,70]: electron-donating and electron-withdrawing groups increase or lower the HOMO energy, respectively. It is worth mentioning that often the surface functionalization does not affect the optical bandgap of CDs, but rather shifts the HOMO/LUMO energies simultaneously. The HOMO energy can be lowered down to −6.80 eV, which was observed for CDs with an oxygen rich surface [55]. A similar effect on the HOMO energy was observed for CDs via doping with halogen atoms [71]. For more details on CD synthetic procedures, interested readers are referred to the recent reviews [72,73,74], including syntheses of pH-sensitive [8] and metal-doped [75] CDs.

### 2.2. Fabrication of CD-Based Solid-State Composites

Further utilization of CDs in devices requires a high PL QY in the solid state. Similar to other nanoparticles, emission of CDs tends to experience a self-quenching in solid state, owing to aggregation. To prevent this undesirable effect, the surface of CDs must be passivated in order to immobilize/separate nanoparticles and retain their emissive properties [13,76]. To achieve this goal, CDs were embedded in different organic and inorganic matrices, such as polymers [15,21,39,77], zeolites [78], potash alum [79], epoxy resin [56,80,81,82], organically modified sol–gel glasses (ORMOSIL) [83,84], starch [63,85], silica [86,87,88,89,90], etc. The use of these matrices not only can preserve optical properties of CDs, but also produce composite materials promising for optoelectronic devices.

**Figure 2:**
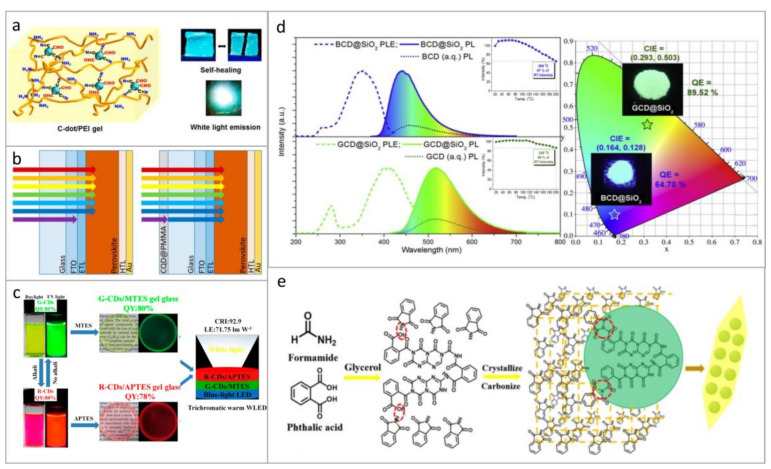
Examples of light-emitting composites based on CDs. (**a**) Model of the gel structure based on CDs incorporated into the PEI framework, and photographs of luminescent CDs-PEI films. Reprinted with permission from reference [91], Copyright American Chemical Society, 2019. (**b**) Schematics of perovskite solar cells without (left) and with (right) light harvesting PMMA layer with embedded CDs. Reprinted with permission from reference [26]. (**c**) Photographs (taken under daylight and UV excitation) of green- and red-emissive CDs in solution and those embedded into MTES or APTES matrices to form CD-based glasses for trichromatic white light emitting diodes (WLEDs). Reprinted with permission from reference [84], Copyright American Chemical Society, 2018. (**d**) PLE and PL spectra of BCD@SiO_2_ and GCD@SiO_2_ composites, with insets showing the temperature dependence of PL spectra; emission color coordinates of these two materials on a CIE 1931 diagram are given on the right-hand side. Reprinted with permission from reference [93], Copyright MDPI AG, 2020 (**e**) Illustration of the synthesis of a composite of CDs and phthalimide crystals. Reprinted with permission from reference [94], Copyright Elsevier, 2020.

The choice of a specific matrix for CDs depends on particular applications. Advantages of polymers are their high transmission in the visible range, advantageous film-forming properties, charge transport ability, and the ease of processing. For example, flexible multicolor emissive films were formed by introducing phenylenediamine-based CDs into polymer poly(vinyl alcohol) (PVA) [15]. Moreover, it was shown that white emission from such hybrid films could easily be realized by mixing red, green, and blue CDs in the appropriate proportions. In Ref. [77], films based on microstructured patterns of CDs in PVA were demonstrated as perspective down-conversion phosphors. The incorporation of CDs into PVA followed by annealing led to a decrease of the oxygen content in the composites, and chemical bonding between the components resulted in their room temperature phosphorescence [21]. Multicolor-emissive CDs based on phenylenediamine were synthesized by Li et al. [39] and mixed with PVA to obtain luminescent transparent films with PL QY as high as 55%, 41%, and 16% for blue, green, and red CDs, respectively. In [91], fabrication of multicolor emissive films from the CD gels incorporated into polyethyleneimine (PEI) network was demonstrated (Figure 2a). These composite materials had high mechanical strength and self-healing properties owing to the efficient bonding between aldehydes at the CD surface and primary amines of PEI via dynamic covalent imine bonds.

In several types of optoelectronic devices, poly(N-vinyl carbazole) (PVK) has been commonly used as a host material for CDs due to its excellent film-forming and hole-transporting properties, and its own PL at 390–410 nm, which can be reabsorbed by CDs [52,60,70]. In [70], blue, green, yellow, and red emissive thin films based on CDs blended with PVK showed high values of PL QY of 56%, 62%, 48%, and 42%, respectively. Poly(methyl methacrylate) (PMMA) was also often used as a matrix for the fabrication of CD-based luminescent films and for fabrication of LEDs and WLEDs [9,55]. In [26], light-harvesting properties of perovskite solar cells with CDs dispersed in the PMMA layer were improved through the down-conversion effect (Figure 2b). In the electroluminescent LED, PMMA can play a role of an isolating layer, which helps to restrain the electron injection from an active CD-containing layer to the hole transport layer, and prevent charge recombination at the anode [92]. As a result, in the presence of the PMMA layer the intensity of blue emission from CDs increased, which changed the LED color from green-yellow to white.

Epoxy resin is frequently used for fabrication of down-conversion LEDs. In [56], CDs with different emission colors were prepared and embedded into epoxy resin to prevent their aggregation while using them as active layer for WLEDs. In [80], it was shown that the emission of WLEDs could be tuned from cold white to warm white by changing the amount of CDs in the epoxy resin.

For blends based on CDs and polymers, it is important to understand how the polarity of the polymer may affect the optical properties of CDs. To address this, Ren et al., investigated CDs embedded into two types of polymers (polystyrene (PS) and PMMA) and into four types of ORMOSIL gel glasses [83]. All studied composites based on CDs had high PL QY of up to 36%, and with increasing polarity of the host, absorption and PL bands of CDs underwent a bathochromic shift. Glasses prepared from methyltriethoxysilane (MTES) or 3-aminopropyltriethoxysilane (APTES) have also been utilized as matrices for CDs in Ref. [84]; transparent green- and red-emitting CD-based composites had high PL QYs of almost 80%, and were successfully used to fabricate trichromatic warm WLEDs (Figure 2c).

To avoid self-absorption in the CD ensembles, Li et al. [95] suggested passivation of CD surface by metal ions, for example, sodium. In [63] such passivation led to an increase of the PL; to further protect CDs from aggregation authors used starch to prepare an orange-emitting powder with a PL QY of 23%. In [85] it was demonstrated that choosing the adequate ratio between CDs and starch particles effectively prevented self-quenching and resulted in a significant increase of the PL QY compared with bare CDs deposited on a glass. Another materials used as a matrix for CDs are metal–organic framework (MOF): Zr(IV)-based MOF was used to design CD-based composites with white emission and high PL QY reaching 37% [96].

Among inorganic matrices, silicon-based ones have been widely used. Zhang et al. [93] presented a composite material based on CDs incorporated into SiO_2_ nanospheres, which showed both improved PL intensity and thermal stability (Figure 2d). It was reported that CDs form covalent bonds with the silica matrix, which facilitated the rise of the phosphorescent signal from those composites [86,87,89]. Incorporation into silica matrices not only prevented CDs from aggregation-caused PL quenching [88] but also increased their PL QY up to 70% [90]. CDs were incorporated into sodium-borosilicate porous glasses [97]; such matrix prevented CDs from aggregation and PL quenching resulting in preservation and even enhancement of their PL QY. Sodium silicate was used as transparent low-toxic matrix to form CD-based 3D glassy-like material with PL emission tunable over a wide spectral range from 450 to 630 nm [98,99]. As reported in [98], the PL QY of these solid samples was even higher than in respective solutions: 30–40% versus 8–32%. In [100], highly luminescent xerogels with PL QY of 40% were fabricated using CDs and tetraethyl orthosilicate (TEOS) through linkage of TEOS molecules and hydroxyl groups at the CD’s surface.

Another approach to fabricate solid-state hybrid materials based on CDs is to perform cocrystallization in a saturated solution of the precursor of matrix of interest [101,102]. In [94], a composite material based on CDs dispersed in phthalimide crystals was prepared by one-step solvothermal synthesis. The synthesis was carried out by using phthalic acid, which acted as a carbon source and at the same time reacted with amino compounds with the formation of phthalimide (Figure 2e), which, in turn, could grow into crystals during the reaction. The resulting composite had a strong emission in solid state. Another method for the formation of hybrid strongly-emitting material based on CDs is described in [103]: a BaSO_4_ shell was formed around CDs and protected them from external factors (high temperature, different solvents, and pH). The synthesized CD-based material had a PL QY of 27% in the solid state.

Thus, implementation of different host matrices for CDs may preserve and even improve their optical responses, which are of great importance for further fabrication of optoelectronic devices, including solar cells and LEDs, as will be considered in the next sections.

## 3. CD-Based Solar Cells

Implementation of CDs in different kinds of solar cells is a popular research direction currently [25,104,105,106]. CDs have been used in different functional layers of solar cells: electron-transporting layers, active absorbing layers, hole-transporting layers, and as an interlayer spacing employed to align and adjust the energy levels of other components.

### 3.1. CDs in Dye-Sensitized Solar Cells

CDs have been used as components of dye-sensitized solar cells (DSSCs) in several possible ways: (i) as a sensitizer, (ii) to modify the photoanode, and (iii) to decorate the counter electrode. Gao et al. used CDs to reduce the HOMO–LUMO gap in order to decrease the rate of charge recombination and to improve charge transfer in DSSCs [25]. On the other hand, it was reported that the addition of CDs might lead to a significant decrease in efficiency of N719 based DSSCs due to the energy transfer between the N719 dye and CDs [107]. In [108], the authors used CDs derived from rosemary leaves and used them to improve the TiO_2_ photoanode, which resulted in a more effective charge carrier separation and filling deep trap sites, which, in turn, increased the electron transfer rate and short-circuit current (J_sc_) of the DSSC, leading to 2.2-fold increase in the power conversion efficiency (PCE). Another example of the modification of the TiO_2_ electrode with CDs was described in Ref. [109] where the increase in the surface area of hybrid CD-TiO_2_ material helped to achieve better adsorption of dye molecules, and thus improved the PCE of solar cells. A ZnO photoanode was modified with nitrogen-rich CDs [110], which resulted in the improvement of charge separation in DSSCs and an increase in the PCE. The latter was determined by several factors: addition of CDs increased the active surface area of ZnO, which resulted in better adsorption of the dye molecules; the bandgap of ZnO decreased and, consequently, the efficiency of charge separation increased. Moreover, amine surface groups of CDs played the role of the electron donor to ZnO, and therefore CDs with high amine content were beneficial to use.

### 3.2. CDs in Perovskite Solar Cells

**Figure 3:**
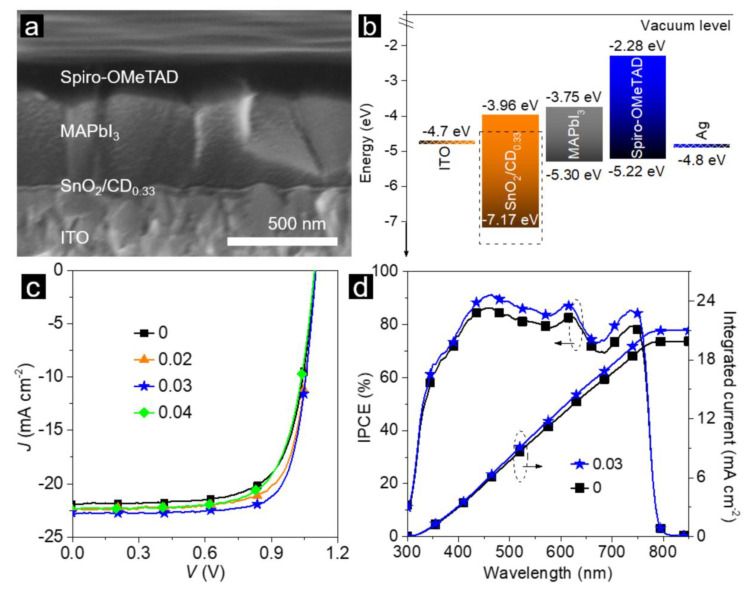
MAPbI_3_ perovskite solar cell (PSC) modified with CDs: (**a**) cross-sectional SEM image; (**b**) energy level band diagram; (**c**) current density (*J*) vs. voltage (*V*) of solar cells with and without (“0”) CD addition; and (**d**) incident photon-to-current efficiency (IPCE) spectra of devices with (“0.03”) and without (“0”) CD addition. Reprinted with permission from reference [111], Copyright Wiley-VCH, 2020.

Several recent reviews were devoted to perovskite solar cells (PSCs) whose performance was improved by carbon nanomaterials [104,106]. CDs have been used in order to improve charge transport characteristics, PCE, and stability of the devices. In [111], CDs were used as an intermediate layer to improve PCE of PSCs from 17.3% to 19.5%, which was determined by reduction of trap states in the perovskite active layer, and the enhancement of the electron extraction capability. Morphological and optoelectronic characteristics of PSCs based on methylammonium (MA)PbI_3_ with added CDs are presented in Figure 3.

CDs were often used to modify electron-transporting layers in PSCs [95,112,113]. Addition of a small amount of CDs into MA iodide solution allowed the fabrication of higher-quality perovskite films, reducing crystal defects, and amount of trap states, therefore non-radiative recombination centers, thus the CD-modified devices showed an increase in PCE from 16.21% to 19.17%, and a better stability [112]. To obtain perovskite films with larger grain size, Guo et al. deposited them onto a sodium-ion-functionalized CDs layer [95]. This resulted in improvement of electron conductivity and reduction of charge carrier recombination, and hence in an increase in average PCE of over 20%. Doping of SnO_2_ by CDs led to an increase in electron mobility by almost 20 times together with improvement of their stability against humidity [113].

CDs were also used in the hole-transporting layers of PSCs. It was shown that composite based on CDs and graphene oxide (GO) can replace poly(3,4-ethylenedioxythiophene) polystyrene sulfonate (PEDOT:PSS) layer in planar-heterojunction PSCs [114]. In contrast to the pure GO as a hole-transporting layer, in the presence of the optimum content of CDs on GO a reduction of the charge recombination rate and more favorable energy level alignment were achieved. The combination of such hole-transporting layer and downshifting layer based on CDs allowed to reach a PCE of 16.8%, and to achieve long term stability of the devices.

## 4. CDs in LEDs and WLEDs

LEDs based on CDs belong to two main types—down-conversion and electroluminescent (charge-injection) ones [115]. The first type can easily be produced by deposition of CDs onto a commercial UV-emitting chip, which serves as an excitation source; they are often mentioned in the literature as a kind of demonstration device with an emission tunable over the wide spectral range, including WLEDs with different color temperatures. While CDs can also be deposited directly from solution, the incorporation of CDs in different matrices, as was discussed in Section 2.2 of this review, provides an opportunity to prevent self-quenching of emission, and thus to achieve a better device performance. It should be emphasized that the availability of red-emitting CDs with high PL QY is still a bottleneck for down-conversion CD-based WLEDs; that is why it is advantageous to combine blue or green-emitting CDs with other red luminophores, such as quantum dots (QDs) [116], rare-earth containing phosphors [81], etc.

In the second type of LEDs, the CDs act as an active layer, where electroluminescence generation occurs through the charge injection by applied electric field. In this regard, there are several restrictions imposed on materials used in such LEDs: they should possess certain positions for the HOMO–LUMO levels; proper selection of electron-/hole-transporting layers in accordance with their energy structure; requirement for high rate of radiative recombination combined with elimination or reduction of nonradiative transitions, etc. Thus, to produce electroluminescent LEDs is much more complicated than to make down-conversion ones, and the efficiency of those LEDs based on CDs is still much lower than their competitors, namely LEDs based on semiconductor QDs, perovskites, or organic molecules.

### 4.1. Down-Conversion CD-Based WLEDs

**Figure 4:**
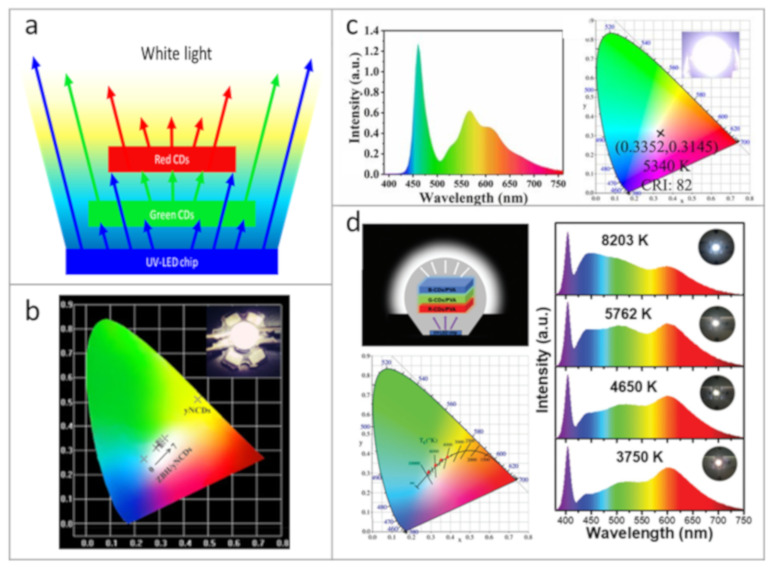
Examples of down-conversion CDs-based WLEDs. (**a**) Scheme of the down-conversion process to generate white light. (**b**) Commission International d’Eclairage (CIE) coordinates of WLEDs based on CDs and zinc borate; the inset shows a photograph of a WLED. Reprinted with permission from reference [117], Copyright Elsevier, 2020. (**c**) Emission spectrum and CIE coordinates of a WLED based on composite material of CDs embedded into phthalimide crystals. Reprinted with permission from reference [94]. (**d**) Illustration of a multi-layered trichromatic WLED; CCT, CIE coordinates, emission spectra, and photographs of WLEDs based on CDs synthesized from o-phenylenediamine. Reprinted with permission from reference [39], Copyright Royal Society of Chemistry, 2020.

Down-conversion WLEDs based on CDs are often mentioned in scientific literature as a kind of prototype devices for the white light emission. The down-conversion approach to generate white light is based on absorption of UV or blue light emitted from commercial LED chip (semiconductor LED based on InGaN, GaN, etc.), which results in the re-emission of light with longer wavelengths (green and red), as presented in Figure 4a. In Ref. [117], composites with white emission based on CDs embedded into zinc borate matrix were used for fabrication of WLEDs by deposition of these CD-based composites onto the commercial UV-LED chips, with Commission International d’Eclairage (CIE) color coordinates shown in Figure 4b. By changing the ratio between CDs and zinc borate, white light temperature could be controlled. Zheng et al. [94] demonstrated a composite material made of CDs embedded into phthalimide crystals with a broad PL band, which allowed them to fabricate WLEDs with color rendering index (CRI) of 82, correlated color temperature (CCT) of 5340 K, and CIE coordinates of (0.3352, 0.3145), which is close to pure white light (Figure 4c). Warm WLEDs with high CRI of 92.9 and luminous efficiency (LE) of 71.75 lm W^−1^ were made by deposition of the two CDs types incorporated in ORMOSIL onto the UV-LED chip [84]. By combination of three layers emitting three basic colors (red, green, and blue) from CDs synthesized from o-phenylenediamine, WLEDs with tunable values of CCT and high CRIs reaching 96.5 were obtained [39], as demonstrated in Figure 4d. By varying the mass content of CDs, the color of resulting WLEDs was tuned, and it was possible to accurately adjust the WLED’s emission in a wide spectral range from warm to cold white light. In [63], the authors presented the fabrication of warm WLEDs based on CDs passivated by sodium ions and then incorporated into starch. The fabricated device had CIE of (0.41, 0.45) and CCT of 3708 K. Wang et al. [62] embedded CDs emitting three basic colors (red, green, and blue) into a PMMA matrix and deposited them onto UV-LED chips, resulting in down-conversion WLEDs with a luminous efficacy of 31.3 lm W^−1^ and CRI of 93. The group of Tian [98] presented down-conversional LEDs with emission tuned over a wide spectral range of 450–640 nm and respective WLEDs. Multicolored CDs synthesized from citric acid and urea in 3 different solvents were mixed with sodium silicate and polydimethylsiloxane to form a luminescent solid material. Green- and red-emissive CD-based composites were deposited onto a UV-LED chip to achieve the WLED with CRI, CCT, CIE coordinates, and LE of 82.4, 5048 K, (0.34, 0.31), and 8.34 lm W^−1^, respectively. The same group also reported the set of WLEDs with emission tuned from cold to warm white light using the mixture of mentioned CDs in sodium silicate in different proportions [99]. Zhou et al. [118] demonstrated WLEDs based on CDs where the optical transitions originated from surface states were enhanced by hydrogen peroxide treatment, which allowed us to achieve WLEDs with following parameters: CIE coordinates of (0.33, 0.34), CCT of 5129 K, and CRI of 79. Recently, Meng et al., reported high LE of 42 lm W^−1^ for WLED based on CDs embedded into graphitic carbon nitride (g-C_3_N_4_) through 2-steps microwave-assisted synthesis [104].

### 4.2. Electroluminescent CD-Based LEDs

**Figure 5:**
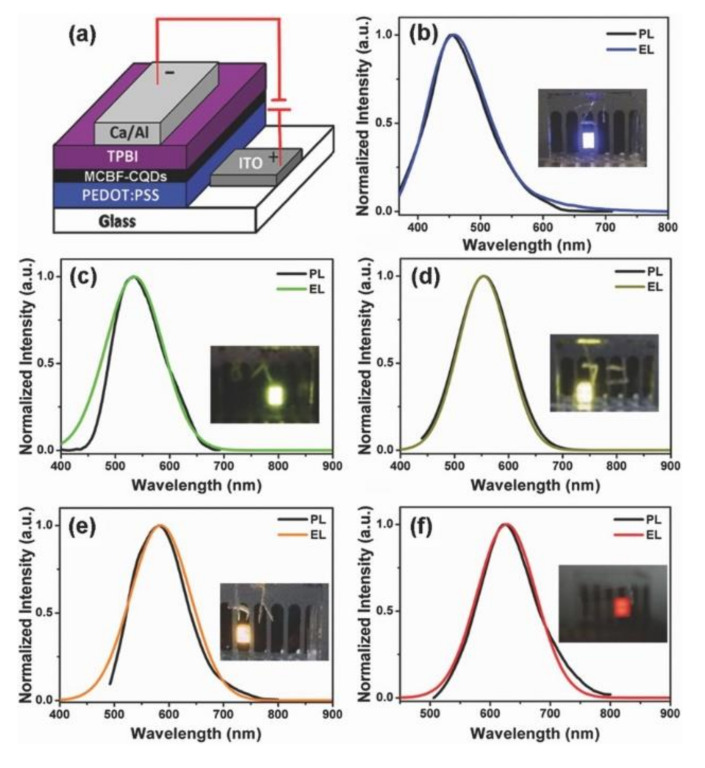
Examples of the multicolor electroluminescent CDs-based LEDs. (**a**) The device structure comprising ITO/PEDOT:PSS (anode), MCBF-CQDs (multicolor bandgap fluorescent carbon quantum dots as an active emission layer), TPBi (ETL), and Ca/Al (cathode). The normalized PL spectra and the corresponding EL spectra of (**b**) blue CDs, (**c**) green CDs, (**d**) yellow CDs, (**e**) orange CDs, and (**f**) red CDs based thin films. The photographs in the insets of (**b**–**f**) display the emission from blue, green, yellow, orange, and red emission of the respective monochrome LEDs. Reprinted with permission from reference [52], Copyright Wiley-VCH, 2017.

In one of the earliest reports on the CD-based electroluminescent LEDs, Fu et al. [41] reported WLEDs with a maximum external quantum yield (EQE) of 0.083%. In recent years a big step forward has been taken in this research area, and the efficiency of such devices has increased to 4% [60], as will be discussed in this section. Zhang et al. [119] reported CD-based LEDs with CDs whose spectral parameters were voltage-dependent, and demonstrated white-color electroluminescence at high voltage. It was discussed how the excitation-dependent PL of the used CDs possessing at least three different relaxation channels with specific PL lifetimes translates into different color of LED emission, which depends on the injection current density. In [55], it was emphasized that the prerequisite for the fabrication of the efficient LEDs is the employment of CDs with high PL QY. The best performing LEDs in that work had a maximum luminance at about 80 cd m^−2^ and EQE of 0.6%. Yuan et al. [52] were among the first to fabricate high-performance monochrome CD-based LEDs with the emission color ranging from blue all the way to the red (Figure 5a), furthermore WLEDs with CIE coordinates of (0.30, 0.33), which is close to the pure white light were also demonstrated. The maximum luminance of these WLEDs reached 2050 cd m^−2^ and current efficiency was 1.1 cd A^−1^. They used CDs blended with PVK as an emissive layer; PL of PVK in the range of 390–410 nm could be absorbed by CDs, and thus energy was efficiently transferred to CDs resulting in the emission enhancement. In [70], the authors used triangular CDs produced from phloroglucinol in various solvents to fabricate blue, green, yellow, and red LEDs with high color-purity and narrow full width and half maxima of 30, 32, 38, and 39 nm, respectively. Green LEDs had a stable performance with a maximum luminance of 4762 cd m^−2^ and current efficiency of 5.11 cd A^−1^. Recently, Sargent et al. [60] presented deep-blue CD-based LEDs with a remarkable EQE of 4%; such a high EQE value was achieved by incorporating CDs into a PVK matrix, which offered favorable film-forming features and prevented the aggregation-induced quenching. Furthermore, the use of poly(9,9-dioctylfluorene-co-N-(4-(3-methylpropyl))diphenylamine) (TFB) as a hole-transporting layer facilitated the charge injection into the active CD layer.

An interesting approach towards electroluminescent WLEDs has been reported in [92]. Nearly white electroluminescence was achieved by combination of the green-yellow emissive ZnO nanowires with blue emissive CDs. The use of an isolating PMMA layer as a part of the structure helped to restrain the electron injection from the active CD-containing layer to hole-transporting layer and to prevent the charge recombination at the anode. As a result, in the presence of PMMA, the blue emission of CDs increased, which altered the LED emission color from green-yellow to white.

To conclude the Section 4, we summarized the recent literature data on CD-based WLEDs in Table 1, which includes both the synthesis conditions for the CDs, the matrices used, the emission color of CDs, and the performance characteristics of the respective WLEDs.

## 5. Conclusions and Outlook

The use of CDs in the field of optoelectronics is of great interest currently and has a high development potential. Alongside well-recognized advantages of CDs as cheap, non-toxic, environmentally friendly materials, a wide variety of the available synthetic methods opens up an opportunity to produce CDs with the desired optical and electrical properties. To date, CDs have been already used in solar cells and LEDs, while the performance of those devices still needs to be further improved. We showed that CDs could improve the PCE of solar cells due to more efficient light-harvesting and charge separation; this was also complemented by improvements of stability. To achieve this goal, both synthetic and post-synthesis procedures should be further developed to precisely control the energy structure, i.e., HOMO/LUMO energy level positions, and to increase the absorption cross-sections in the spectral region of interest. The former can be achieved by increasing the size of sp^2^-hybridized domains within CDs, heteroatom doping, and surface functionalization. The increase of the CD absorption for efficient light-harvesting can be achieved by doping of the CD core with heteroatoms and metals, and by synthesizing particles with suitable morphologies, such as disk-like CDs with oxygen-rich surface. Use of CDs in down-conversion LEDs has been shown to be a perspective direction due to the ease of their fabrication and a wide variety of spectral characteristics of CD-based solid-state composites. We summarized the recent synthetic efforts aimed at obtaining CDs with strong emission in the red spectral region, and the fabrication procedures towards CD-based composites with stable optical responses. Another direction in the LED development, promising but yet to be developed, is the fabrication of electroluminescent CD-based LEDs, which demands precise control of electronic structure, high efficiency of charge injection, and high QY with the possibility of controlled color tuning. In conclusion, this review will be useful for both scientists studying the fundamental properties of CDs and researchers engaged in development of CD-based optoelectronic and photonic devices.

## Figures and Tables

**Table 1:** Materials and performance characteristics of CD-based WLEDs.

CD Precursors; Solvent	Synthesis Conditions: Type, Temperature/Power, Time	PL Band Position of CDs, nm	Matrix	CRI	CCT, K	CIE Coordinates	LED’s Luminous Efficiency, lm W^−1^	Ref.
CA and 1-hexadecylamine; dimethylbenzene	solvothermal, 180 °C, 12 h	460	PDMS	>89	6000	(0.335, 0.332), (0.331, 0.328)		[120]
CA and PAN; ethanol	solvothermal, 200 °C, 7 h	375, 450 and 585 (under λ_ex_ 320, 350 and 470 nm)	epoxy resin	82.4		(0.29, 0.31)		[56]
o-PD and Tris; H_2_SO_4_ aqueous solution	solvothermal, 220 °C, 10 h	445, 508, or 611	PVA	96.5	4650	(0.362, 0.370)		[39]
urea and CA; water	hydrothermal, 160 °C, 4 h	439				(0.3117, 0.2791)		[121]
CA and EDA or urea; water	hydrothermal, 200 °C, 5 h or microwave-assisted, 650 W, 5 min	440 or 520	silica nano-spheres	89.1	4850	(0.3514, 0.3715)		[93]
CA and EDA; water	hydrothermal, 200 °C, 5 h	456	PMMA	91		(0.32, 0.33)	15.1	[116]
CA urea, NaOH; water	microwave-assisted, 750 W, 3 min	518	epoxy resin	93.5	4333	(0.366, 0.366)	52.3	[81]
o-PD; water	hydrothermal, 160 °C, 5 h		zinc borate			(0.2887, 0.3088)–(0.3235, 0.3500)		[117]
dimethyl trithiocarbonate, HNO_3_; acetone	solvothermal, 220 °C, 10 h	545		88.38	5389	(0.33, 0.30)		[82]
3,4,9,10-Tetranitroperylene and NaOH; ethanol	solvothermal, 200 °C, 12 h.	515 or 610	MTES and APTES	92.9	3610	(0.4046, 0.4028)	71.75	[84]
p-PD and NaOH; ethanol and IPTS	solvothermal, 180 °C, 12h	527 (in toluene) and 619 (in water)	PMMA or APTES–Gel and PS	70; 85	3949; 4494	(0.397, 0.428);(0.385, 0.345)	15.88; 22	[83]
o-PD or p-PD; dimethylformamide	solvothermal, 200 °C, 4 h	550 or 610	PVB	83	3722	(0.3943, 0.3869)	66.17	[51]
l-Aspartic acid; water and ammonia solution	microwave-assisted, 750 W	456	epoxy resin	83	6987	(0.30, 0.35)	1.281	[80]
phthalic acid; formamide and glycerol	solvothermal, 180 °C, 4 h		phthalimide crystals	82	5430	(0.3352, 0.3145)		[94]
1,3-dihydroxynaphthalene and KIO_4_; ethanol	solvothermal, 180 °C, 4 h	630	silicone or PMMA	97	3875	(0.3924, 0.3912)	31.3	[62]
CA and urea;dimethylformamide	solvothermal, 160 °C, 6 h	580	starch		3708	(0.41, 0.45)		[63]
CA and DAN; ethanol/concentrated sulfuric acid	solvothermal, 200 °C, 1, 4 or 9 h	430, 513, 535, 565, and 604	PVK			(0.30, 0.33)	Luminance 2050 cd m^−2^; current efficiency 1.1 cd A^−1^	[52]
urea and CA; water	hydrothermal, 200 °C, 5 h	523	PMMA			(0.30, 0.36)		[92]
CA and urea; ammonia water	microwave-assisted, 700 W, 6 min	450–500	Zr-MOF, thermal-curable silicone resin	82		(0.31, 0.34)	1.7	[96]
CA and urea; water	microwave-assisted, 750 W, 5 min	500–550	TEOS; PDMS	79	5603	(0.33, 0.34)	28	[100]
CA and urea; water or glycerol or DMF	solvothermal, 160 °C. 4 h	450 or 550 or 600	sodium silicate; PDMS	82.4	5048	(0.34,0.31)	8.34	[98]
CA and urea; water or glycerol or DMF	solvothermal, 160 °C. 4 h	450 or 550 or 650	sodium silicate; PDMS	85; 88; 86	9927; 6109; 3510	(0.27, 0.31); (0.32, 0.33); (0.41, 0.41)	7.8; 6.3; 5.2	[99]
CA and urea; water	microwave-assisted, 750 W, 3–4 min	520	graphitic carbon nitride (g-C_3_N_4_); epoxy resin		7557	(0.29, 0.33)	42	[102]
CA; ammonia water; followed by hydrogen-peroxide-treatment	microwave-assisted, 650 W, 5 min	435	PDMS	79	5240	(0.34, 0.37)		[118]

Abbreviations: CA—citric acid, PDMS—polydimethylsiloxane, PAN –(2-pyridylazo)-2-naphthol, PD—phenylenediamine, Tris—tris(hydroxymethyl)aminomethane, PVA—poly(vinyl alcohol), EDA—ethylenediamine, PMMA—poly(methyl methacrylate), MTES—methyltriethoxysilane, APTES—3-triethoxysilylpropylamine, IPTS—3-isocyanatopropyltriethoxysilane, PS—polystyrene, PVB—polyvinyl butyral, DAN—diaminonaphthalene, PVK—poly(N-vinyl carbazole), MOF—metal–organic framework, TEOS—tetraethyl orthosilicate, PDMS—polydimethylsiloxane.
